# Supplementary material for: Allosteric regulation and crystallographic fragment screening of SARS-CoV-2 NSP15 endoribonuclease
Source: Nucleic Acids Res. 2023 Apr 28;51(10):5255–70. doi: 10.1093/nar/gkad314 (PMC10250223; doi:10.1093/nar/gkad314)
Supplement: gkad314_Supplemental_Files [file gkad314_supplemental_files.zip › Supplementary datasets.zip!/188/Refine_9/01-BUSTER/Cycle-3/shell.01/R_Rfree.html]

# Rxpct factors in resolution bins at cycle 0


|  |  |  |  |  |  |  |  |  |
| --- | --- | --- | --- | --- | --- | --- | --- | --- |
|  | **<d> (Å)**  Rxpct\_Work | Rxpct\_Free | Rxpct\_All | |  |  |  |  |
|  | 16.0655 | 0.3852 | 0.4927 | 0.3887 |  |
|  | 9.2754 | 0.2232 | 0.2713 | 0.2260 |  |
|  | 7.1847 | 0.1986 | 0.2387 | 0.2009 |  |
|  | 6.0722 | 0.2037 | 0.2511 | 0.2058 |  |
|  | 5.3552 | 0.1598 | 0.2705 | 0.1644 |  |
|  | 4.8439 | 0.1492 | 0.2148 | 0.1531 |  |
|  | 4.4558 | 0.1451 | 0.2071 | 0.1483 |  |
|  | 4.1481 | 0.1511 | 0.2055 | 0.1542 |  |
|  | 3.8965 | 0.1573 | 0.2284 | 0.1607 |  |
|  | 3.6857 | 0.1676 | 0.2516 | 0.1714 |  |
|  | 3.5058 | 0.1718 | 0.2080 | 0.1736 |  |
|  | 3.3499 | 0.1788 | 0.2358 | 0.1821 |  |
|  | 3.2131 | 0.1917 | 0.2157 | 0.1931 |  |
|  | 3.0918 | 0.1917 | 0.2494 | 0.1943 |  |
|  | 2.9833 | 0.1940 | 0.2404 | 0.1960 |  |
|  | 2.8855 | 0.2052 | 0.2431 | 0.2072 |  |
|  | 2.7967 | 0.2054 | 0.2517 | 0.2075 |  |
|  | 2.7156 | 0.2105 | 0.2343 | 0.2117 |  |
|  | 2.6412 | 0.2669 | 0.2615 | 0.2665 |  |
|  | 2.5725 | 0.3543 | 0.2683 | 0.3514 |  |
|  | **Overall** | 0.1871 | 0.2406 | 0.1898 |  |

# Rxpct factors in resolution bins at cycle 1


|  |  |  |  |  |  |  |  |  |
| --- | --- | --- | --- | --- | --- | --- | --- | --- |
|  | **<d> (Å)**  Rxpct\_Work | Rxpct\_Free | Rxpct\_All | |  |  |  |  |
|  | 16.0655 | 0.3873 | 0.4957 | 0.3908 |  |
|  | 9.2754 | 0.2237 | 0.2721 | 0.2265 |  |
|  | 7.1847 | 0.1989 | 0.2382 | 0.2011 |  |
|  | 6.0722 | 0.2032 | 0.2507 | 0.2053 |  |
|  | 5.3552 | 0.1591 | 0.2718 | 0.1638 |  |
|  | 4.8439 | 0.1485 | 0.2146 | 0.1524 |  |
|  | 4.4558 | 0.1445 | 0.2064 | 0.1477 |  |
|  | 4.1481 | 0.1507 | 0.2068 | 0.1539 |  |
|  | 3.8965 | 0.1571 | 0.2279 | 0.1605 |  |
|  | 3.6857 | 0.1678 | 0.2520 | 0.1715 |  |
|  | 3.5058 | 0.1721 | 0.2100 | 0.1740 |  |
|  | 3.3499 | 0.1789 | 0.2370 | 0.1822 |  |
|  | 3.2131 | 0.1919 | 0.2153 | 0.1933 |  |
|  | 3.0918 | 0.1921 | 0.2490 | 0.1947 |  |
|  | 2.9833 | 0.1932 | 0.2387 | 0.1951 |  |
|  | 2.8855 | 0.2047 | 0.2429 | 0.2066 |  |
|  | 2.7967 | 0.2049 | 0.2503 | 0.2069 |  |
|  | 2.7156 | 0.2091 | 0.2330 | 0.2103 |  |
|  | 2.6412 | 0.2654 | 0.2621 | 0.2652 |  |
|  | 2.5725 | 0.3530 | 0.2585 | 0.3499 |  |
|  | **Overall** | 0.1869 | 0.2408 | 0.1896 |  |

# Rxpct factors in resolution bins at cycle 2


|  |  |  |  |  |  |  |  |  |
| --- | --- | --- | --- | --- | --- | --- | --- | --- |
|  | **<d> (Å)**  Rxpct\_Work | Rxpct\_Free | Rxpct\_All | |  |  |  |  |
|  | 16.0655 | 0.3874 | 0.4942 | 0.3910 |  |
|  | 9.2754 | 0.2232 | 0.2711 | 0.2260 |  |
|  | 7.1847 | 0.1979 | 0.2376 | 0.2002 |  |
|  | 6.0722 | 0.2019 | 0.2482 | 0.2039 |  |
|  | 5.3552 | 0.1585 | 0.2711 | 0.1631 |  |
|  | 4.8439 | 0.1475 | 0.2141 | 0.1515 |  |
|  | 4.4558 | 0.1442 | 0.2063 | 0.1474 |  |
|  | 4.1481 | 0.1506 | 0.2081 | 0.1540 |  |
|  | 3.8965 | 0.1570 | 0.2278 | 0.1604 |  |
|  | 3.6857 | 0.1673 | 0.2519 | 0.1711 |  |
|  | 3.5058 | 0.1723 | 0.2093 | 0.1742 |  |
|  | 3.3499 | 0.1787 | 0.2361 | 0.1820 |  |
|  | 3.2131 | 0.1921 | 0.2150 | 0.1934 |  |
|  | 3.0918 | 0.1920 | 0.2494 | 0.1946 |  |
|  | 2.9833 | 0.1934 | 0.2386 | 0.1953 |  |
|  | 2.8855 | 0.2050 | 0.2432 | 0.2069 |  |
|  | 2.7967 | 0.2053 | 0.2497 | 0.2073 |  |
|  | 2.7156 | 0.2102 | 0.2351 | 0.2115 |  |
|  | 2.6412 | 0.2661 | 0.2615 | 0.2658 |  |
|  | 2.5725 | 0.3516 | 0.2648 | 0.3488 |  |
|  | **Overall** | 0.1867 | 0.2405 | 0.1894 |  |

# Rxpct factors in resolution bins at cycle 3


|  |  |  |  |  |  |  |  |  |
| --- | --- | --- | --- | --- | --- | --- | --- | --- |
|  | **<d> (Å)**  Rxpct\_Work | Rxpct\_Free | Rxpct\_All | |  |  |  |  |
|  | 16.0655 | 0.3890 | 0.4952 | 0.3925 |  |
|  | 9.2754 | 0.2208 | 0.2781 | 0.2242 |  |
|  | 7.1847 | 0.1952 | 0.2340 | 0.1974 |  |
|  | 6.0722 | 0.1961 | 0.2465 | 0.1983 |  |
|  | 5.3552 | 0.1566 | 0.2754 | 0.1615 |  |
|  | 4.8439 | 0.1460 | 0.2139 | 0.1500 |  |
|  | 4.4558 | 0.1421 | 0.2037 | 0.1453 |  |
|  | 4.1481 | 0.1491 | 0.2095 | 0.1525 |  |
|  | 3.8965 | 0.1568 | 0.2314 | 0.1603 |  |
|  | 3.6857 | 0.1669 | 0.2490 | 0.1705 |  |
|  | 3.5058 | 0.1727 | 0.2124 | 0.1746 |  |
|  | 3.3499 | 0.1772 | 0.2378 | 0.1806 |  |
|  | 3.2131 | 0.1913 | 0.2180 | 0.1928 |  |
|  | 3.0918 | 0.1912 | 0.2523 | 0.1940 |  |
|  | 2.9833 | 0.1935 | 0.2339 | 0.1952 |  |
|  | 2.8855 | 0.2048 | 0.2402 | 0.2066 |  |
|  | 2.7967 | 0.2047 | 0.2475 | 0.2066 |  |
|  | 2.7156 | 0.2115 | 0.2426 | 0.2130 |  |
|  | 2.6412 | 0.2626 | 0.2641 | 0.2627 |  |
|  | 2.5725 | 0.3472 | 0.3016 | 0.3457 |  |
|  | **Overall** | 0.1854 | 0.2414 | 0.1882 |  |
